# Synthesis and characterization of TADDOL-based chiral group six PNP pincer tricarbonyl complexes

**DOI:** 10.1007/s00706-018-2281-0

**Published:** 2018-10-20

**Authors:** Sara R. M. M. de Aguiar, Christian Schröder-Holzhacker, Jan Pecak, Berthold Stöger, Karl Kirchner

**Affiliations:** 1 Institute of Applied Synthetic Chemistry, Vienna University of Technology, Getreidemarkt 9, 1060 Vienna, Austria; 2 X-ray Center, Vienna University of Technology, Getreidemarkt 9, 1060 Vienna, Austria

**Keywords:** Chiral pincer ligands, Group six metals, Solvothermal synthesis, TADDOL, Carbonyl ligands

## Abstract

**Abstract:**

The new chiral PNP pincer ligand *N*^2^,*N*^6^-bis((3a*R*, 8a*R*)-2,2-dimethyl-4,4,8,8-tetraphenyltetrahydro[1,3]dioxolo[4,5-*e*][1,3,2]dioxaphosphepin-6-yl)pyridine-2,6-diamine (PNP-TADDOL) was synthesized in 80% isolated yield. Complexes of the type [M(PNP-TADDOL)(CO)_3_] (M = Cr, Mo, and W) were prepared via a solvothermal approach. This methodology constitutes a fast, simple, and practical synthetic method to obtain complexes of that type in high isolated yields. The X-ray structure of the molybdenum complex is presented.

**Graphical abstract:**

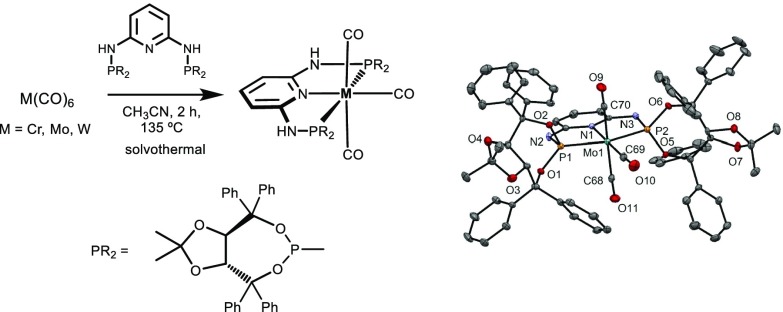

## Introduction

PNP pincer ligands with a pyridine backbone and phosphorus donors connected in the two *ortho* position via CH_2_, O, NH, or NR spacers are widely utilized in transition metal chemistry. They form often very stable but also reactive complexes which can be designed in modular fashion. They typically adopt a meridional coordination mode via the two electron donor groups and a metal–nitrogen bond. Over the last decades, pincer complexes have found numerous applications in various areas of chemistry, especially organic synthesis and catalysis [1–12].

As group six PNP complexes are concerned, such compounds are exceedingly rare and only a few examples have been reported in the literature [13–26]. We are currently focusing on the synthesis and reactivity of group six complexes containing PNP pincer ligands based on the 2,6-diaminopyridine scaffold. In these ligands, the aromatic pyridine ring and the phosphine moieties are connected via NH, *N*-alkyl, or *N*-aryl spacers [27–31]. Such ligands were first utilized by the group of Haupt who prepared PNP pincer complexes of the type [M(PNP-Ph)(CO)_3_] (M = Cr, Mo, W; PNP-Ph = *N*^2^,*N*^6^-bis(diphenylphosphanyl)pyridine-2,6-diamine) [13]. In continuation of our studies on group six PNP complexes, we report here the synthesis of chiral zero valent Cr, Mo, and W PNP pincer complexes based on ((4*R*,5*R*)-2,2-dimethyl-1,3-dioxolane-4,5-diyl)bis(diphenylmethanol) (R,R-TADDOL). It has to be mentioned that chiral pincer complexes are comparatively rare [32–34].

## Results and discussion

The new C_2_-symmetric chiral pincer ligand *N*^2^,*N*^6^-bis((3a*R*,8a*R*)-2,2-dimethyl-4,4,8,8-tetraphenyltetrahydro[1,3]dioxolo[4,5-*e*][1,3,2]dioxaphosphepin-6-yl)pyridine-2,6-diamine (PNP-TADDOL, **2**) was obtained in 80% isolated yield by reacting 2,6-diaminopyridine with 2 equiv. of **1** in the presence of NEt_3_ as base as shown in Scheme 1. The pincer ligand is air-stable and was characterized by ^1^H, ^13^C{^1^H}, and ^31^P{^1^H} NMR spectroscopy.

**Figure:**
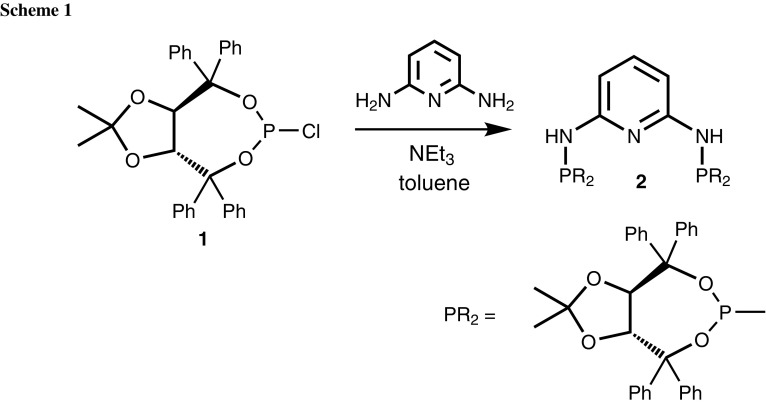

A suspension of the hexacarbonyl complexes M(CO)_6_ (M = Cr, Mo, W) and the PNP ligand **2** in CH_3_CN were placed in a sealed microwave glass vial and stirred for 2 h at 135 °C. After workup, the analytically pure products [M(PNP-TADDOL)(CO)_3_] **3**–**5** could be isolated in 89–91% yields (Scheme 2). All complexes are air stable. Moreover, in the presence of small amounts of water, P–N and/or P–O bond cleavage as a result of hydrolysis was not observed.

**Figure:**
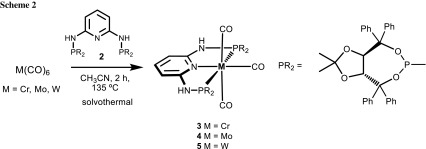

All complexes were fully characterized by a combination of ^1^H, ^13^C{^1^H}, and ^31^P{^1^H} NMR spectroscopy, IR spectroscopy, and elemental analysis. In the ^13^C{^1^H} NMR spectrum, these complexes exhibit two characteristic low-field triplet resonances in a 1:2 ratio in the range of 228–206 ppm assignable to the carbonyl carbon atoms *trans* and *cis* to the pyridine nitrogen atom, respectively (Table 1). The ^31^P{^1^H} NMR spectra exhibit singlet resonances in the range of 198–153 ppm. In the case of the tungsten complex, the spectra exhibit singlet resonances with ^1^*J*_WP_ coupling constants of 499 Hz. The tungsten–phosphorus coupling was detected as a doublet satellite due to ^183^W. This isotope has a 14% natural abundance with a spin *I* of 1/2. This signal is superimposed over the dominant singlet.

**Table 1:** Selected ^13^C{^1^H} and ^31^P{^1^H} NMR and IR data of complexes **3**–**5** and related molybdenum tricarbonyl complexes [13, 15, 16]

Complexes	*δ*_CO_/ppm		*δ*_P_/ppm	*δ*_CO_/cm^−1^		
[Cr(PNP-TADDOL)(CO)_3_] (**3**)	228.0	216.9	197.8	1974	1921	1865
[Mo(PNP-TADDOL)(CO)_3_] (**4**)	221.6	206.7	172.7	1980	1946	1867
[W(PNP-TADDOL)(CO)_3_] (**5**)	214.3	201.0	153.8	1978	1932	1865
[Mo(PNP-BIPOL)(CO)_3_]	224.7	208.4	204.8	1985	1876	–
[Mo(PNP-Ph)(CO)_3_]	228.4	211.2	104.0	1964	1858	1765
[Mo(PNP^Me^-Ph)(CO)_3_]	227.8	211.9	131.0	1956	1911	1850
[Mo(PNP^Me^-Et)(CO)_3_]	230.6	214.9	132.3	1942	1822	1806
[Mo(PNP-Cy)(CO)_3_]	231.1	216.4	122.6	1941	1828	1790
[Mo(PNP-*i*Pr)(CO)_3_]	231.4	216.9	132.7	1936	1809	1790
[Mo(PNP^Me^-*i*Pr)(CO)_3_]	230.8	217.9	159.0	1936	1810	1795
[Mo(PNP-Et)(CO)_3_]	230.3	213.8	111.3	1929	1840	1780
[Mo(PNP-*t*Bu)(CO)_3_]	233.1	224.0	148.8	1922	1808	1771

Both the carbonyl resonances (*δ*_CO_) and the phosphorus resonances (*δ*_P_) exhibit a significant upfield shift on going from Cr to Mo to W. In all complexes, the PNP pincer ligand is coordinated in *mer* fashion with no evidence for any *fac* isomers. As expected of a *mer* CO arrangement, these complexes exhibit the typical three strong to medium absorption bands in the IR spectra. These are assignable to one weaker symmetric and two strong asymmetric vibrations. The *ν*_CO_ frequencies, in particular the symmetric CO stretch, which presents the highest stretching frequency, are indicative for the increasing electron donor strength/decreasing π-acceptor strength of the PNP ligands. When compared to previous results, they follow the order PNP-TADDOL < PNP-BIPOL < PNP-Ph < PNP^Me^-Ph < PNP^Me^-Et < PNP-Cy < PNP-*i*Pr ≈ PNP^Me^-*i*Pr < PNP-Et < PNP-*t*Bu as shown in Scheme 3 and Table 1 [13, 15–17, 27, 31].

**Figure:**
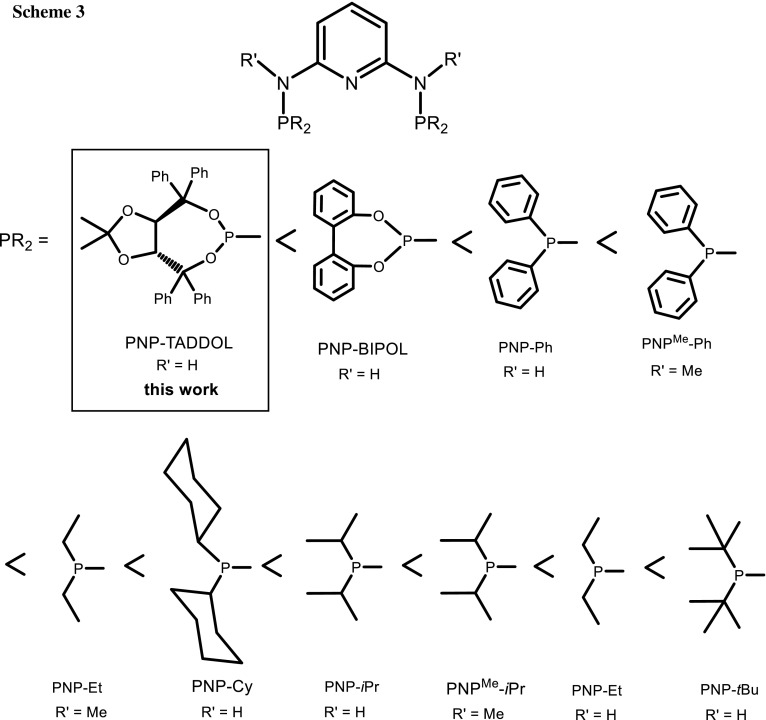

**Fig. 1:**
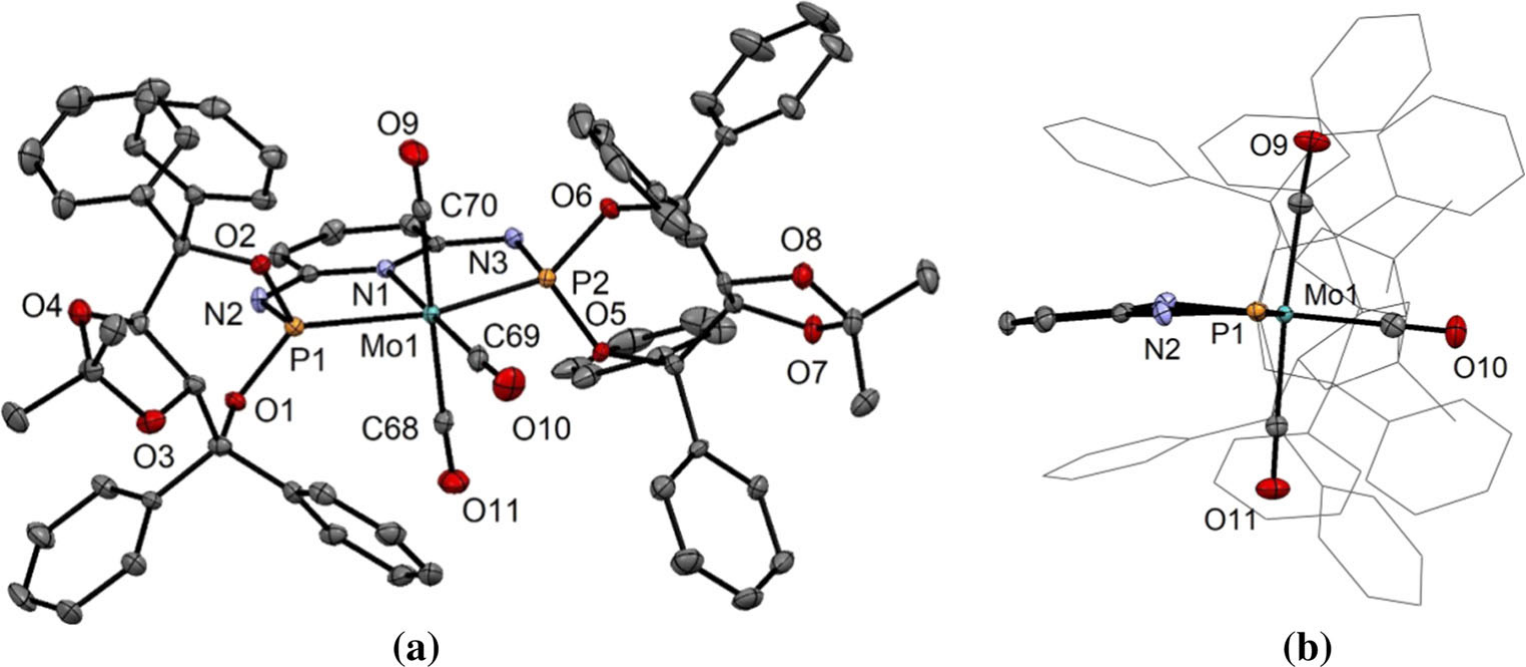
**a** ORTEP plot of [Mo(PNP-TADDOL)(CO)_3_]·**4**CH_2_Cl_2_ (**4**·3CH_2_Cl_2_) showing 50% thermal ellipsoids (hydrogen atoms and solvent omitted for clarity). **b** Side view of **4**·3CH_2_Cl_2_. Selected bond lengths (Å) and bond angles (°): Mo_1_–C_68_ 2.029(6), Mo_1_–C_69_ 1.967(6), Mo_1_–C_70_ 2.037(6), Mo_1_–P_1_ 2.329(1), Mo_1_–P_2_ 2.361(1), Mo–N_1_ 2.242(4), P_1_–Mo_1_–P_2_ 155.61(5), N_1_–Mo_1_–C_69_ 175.3(2), C_68_–Mo_1_–C_70_ 175.8(2)

In addition to the spectroscopic characterization, the solid-state structure of **4** was determined by X-ray crystallography. A molecular view is depicted in Fig. 1 with selected bond distances given in the caption. This complex is best described as distorted octahedron with P–Mo–P and *trans*-C_CO_–Mo–C_CO_ bond angles of 155.61(5)° and 175.8(2)°, respectively. For comparison with results reported previously, in [Mo(PNP)(CO)_3_] complexes with PNP = PNP-TADDOL < PNP-Ph < PNP^Me^-Ph < PNP-*i*Pr < PNP^Me^-*i*Pr < PNP-*t*Bu), the P_1_–Mo–P_2_ angles are also hardly affected by the size of the substituents of the phosphorus atoms, being 155.61(5)°, 155.0(2)°, 155.48(1)°, 155.62(1)°, 155.3(1)°, and 151.73(1)°, respectively. This is a typical feature of pyridine-based PNP pincer ligands. On the other hand, the carbonyl–Mo–carbonyl angles of the CO ligands *trans* to one another deviate significantly from 180° and strongly depend on the bulkiness of the PR_2_ moiety and decrease from 175.8(2)° in [Mo(PNP-TADDOL)(CO)_3_] (**4**) to 171.1(8)° in [Mo(PNP-Ph)(CO)_3_] to 166.15(5)° in [Mo(PNP^Me^-Ph)(CO)_3_] to 166.03(5)° in [Mo(PNP-*i*Pr)(CO)_3_] to 162.93(7)° in [Mo(PNP^Me^-*i*Pr)(CO)_3_], and finally to 156.53(4)° in [Mo(PNP-*t*Bu)(CO)_3_]. Therefore, the steric demand of the PNP pincer ligands increases in the order PNP-TADDOL < PNP-Ph < PNP^Me^-Ph < PNP-*i*Pr < PNP^Me^-*i*Pr < PNP-*t*Bu.

## Conclusion

In summary, we have prepared and fully characterized chiral group six metal complexes of the general formula [M(PNP-TADDOL)(CO)_3_] (M = Cr, Mo, W) bearing the C_2_-symmetric chiral TADDOL-based PNP pincer ligand with a pyridine backbone. Steric and electronic properties of these complexes could be directly compared with a series of other group six metal PNP pincer tricarbonyl complexes. Based on the symmetric CO stretch, which presents the highest stretching frequency, PNP-TADDOL is the weakest σ-donor, but the strongest π-acceptor of all PNP ligands in the series PNP-TADDOL < PNP-BIPOL < PNP-Ph < PNP^Me^-Ph < PNP^Me^-Et < PNP-Cy < PNP-*i*Pr ≈ PNP^Me^-*i*Pr < PNP-Et < PNP-*t*Bu. The X-ray structure of the molybdenum complex [Mo(PNP-TADDOL)(CO)_3_] is presented.

## Experimental

All manipulations were performed under an inert atmosphere of argon using Schlenk techniques or in an MBraun inert-gas glovebox. The solvents were purified according to the standard procedures [35]. The deuterated solvents were purchased from Aldrich and dried over 4 Å molecular sieves. The ligand precursor (3a*R*, 8a*R*)-6-chloro-2,2-dimethyl-4,4,8,8-tetraphenyltetrahydro[1,3]dioxolo[4,5-*e*][1,3,2]dioxaphosphepine (TADDOL-PCl, **1**) was prepared according to the literature [36]. ^1^H, ^13^C{^1^H}, and ^31^P{^1^H} NMR spectra were recorded on Bruker AVANCE-250, AVANCE-400 DRX, and AVANCE-600 spectrometers. ^1^H and ^13^C{^1^H} NMR spectra were referenced internally to residual protio-solvent, and solvent resonances, respectively, and are reported relative to tetramethylsilane (*δ* = 0 ppm). ^31^P{^1^H} NMR spectra were referenced externally to H_3_PO_4_ (85%) (*δ* = 0 ppm).

### *N*^2^,*N*^6^-bis((3a*R*,8a*R*)-2,2-dimethyl-4,4,8,8-tetraphenyltetrahydro[1,3]dioxolo[4,5-*e*][1,3,2]dioxaphosphepin-6-yl)pyridine-2,6-diamine (PNP-TADDOL, **2**, C_67_H_61_N_3_O_8_P_2_)

To a solution of 415 mg 2,6-diaminopyridine (4.11 mmol) in 100 cm^3^ toluene, 1.1 cm^3^ NEt_3_ (10.27 mmol) was added. After cooling to 0 °C, a solution of 4.58 g **1** (8.63 mmol) in 30 cm^3^ of toluene was added and the reaction mixture was stirred for 12 h at 80 °C. The suspension was filtered over Celite and the solvent was removed under reduced pressure. The product was obtained as white powder. The crude product was purified via flash chromatography using silica (conditioned with 5 vol% NEt_3_) and PE/EE (5:1) as eluent. The pure product was obtained as a white powder in 80% yield. ^1^H NMR (CDCl_3_, 20 °C): *δ* = 7.66 (br, 4H, Ph), 7.58 (br, 4H, Ph), 7.38–7.05 (m, 33H, Ph, py^4^), 6.06 (d, ^3^*J*_HH_ = 8.0 Hz, 2H, py^3,5^), 5.50 (d, ^2^*J*_PH_ = 3.9 Hz, 2H, NH), 5.26 (dd, ^3^*J*_HH_ = 8.4 Hz, ^4^*J*_PH_ = 2.8 Hz, 2H, CH^TAD^), 4.86 (d, ^3^*J*_HH_ = 8.5 Hz, 2H, CH^TAD^), 1.21 (s, 6H, CH_3_^TAD^), 0.23 (s, 6H, ${\text{CH}}_{3}^{\text{TAD}}$) ppm; ^13^C{^1^H} NMR (CDCl_3_, 20 °C): *δ* = 154.36 (d, ^2^*J*_CP_ = 17.3 Hz, py^2,6^), 146.12 (Ph), 145.53 (d, ^3^*J*_CP_ = 2.8 Hz, Ph), 141.73 (d, ^3^*J*_CP_ = 1.9 Hz, Ph), 140.95 (d, ^3^*J*_CP_ = 1.8 Hz, Ph), 139.22 (py^4^), 129.02 (Ph), 128.59 (d, *J* = 3.4 Hz, Ph), 128.19 (Ph), 127.80 (Ph), 127.75 (Ph), 127.53 (Ph), 127.45 (Ph), 127.36 (Ph), 127.26 (Ph), 127.15 (d, *J* = 3.2 Hz, Ph), 112.17 (${\text{C(CH}}_{3} )_{2}^{\text{TAD}}$), 100.75 (d, ^3^*J*_CP_ = 13.2 Hz, py^3,5^), 82.95 (C(Ph)_2_), 82.67 (d, ^3^*J*_CP_ = 7.7 Hz, CH^TAD^), 82.48 (d, ^3^*J*_CP_ = 6.0 Hz, CH^TAD^), 82.24 (C(Ph)_2_), 27.49 (${\text{CH}}_{3}^{\text{TAD}}$), 25.32 (${\text{CH}}_{3}^{\text{TAD}}$) ppm; ^31^P{^1^H} NMR (CDCl_3_, 20 °C): *δ* = 134.0 ppm.

### General synthetic procedure for the synthesis of [M(PNP-TADDOL)(CO)_3_] complexes

A suspension of the metal hexacarbonyl (0.60 mmol) and 1 equiv of the PNP ligand **2** (0.60 mmol) in 4 cm^3^ CH_3_CN was placed in a 20 cm^3^ sealed glass tube and stirred for 2 h at 135 °C, whereupon a clear solution was formed. The reaction mixture was allowed to cool to room temperature without stirring. The products were then obtained as crystalline materials and were decanted and washed with *n*-pentane. The solvent was removed under reduced pressure.

#### (*N*^2^,*N*^6^-bis((3a*R*,8a*R*)-2,2-dimethyl-4,4,8,8-tetraphenyltetrahydro[1,3]dioxolo[4,5-*e*][1,3,2]dioxaphosphepin-6-yl)pyridine-2,6-diamine)(tricarbonyl)chromium(0) ([Cr(PNP-TADDOL)(CO)_3_], **3**, C_70_H_61_CrN_3_O_11_P_2_)

The product was obtained as a yellow solid in 91% yield. ^1^H NMR (CD_2_Cl_2_, 20 °C): *δ* = 7.60 (d, *J* = 4.2 Hz, 4H, Ph), 7.46 (d, *J* = 3.3 Hz, 4H, Ph), 7.32 (br, 4H, Ph), 7.25 (br, 2H, Ph), 7.24–7.15 (m, 26H, Ph), 6.88 (t, *J* = 7.4 Hz, 1H, py^4^), 5.33 (dd, ^3^*J*_HH_ = 27.0 Hz, ^4^*J*_PH_ = 7.7 Hz, 4H, CH^TAD^), 5.31 (d, ^3^*J*_HH_ = 7.7, 2H, py^3,5^), 5.11 (s, 2H, NH), 0.55 (s, 6H, ${\text{CH}}_{3}^{\text{TAD}}$), 0.40 (s, 6H, ${\text{CH}}_{3}^{\text{TAD}}$) ppm; ^13^C{^1^H} NMR (CD_2_Cl_2_, 20 °C): *δ* = 228.0 (t, *J* = 13.4 Hz, CO), 216.9 (t, *J* = 20.5 Hz, CO), 156.5 (t, ^2^*J*_CP_ = 13.2 Hz, py^2,6^), 144.2 (Ph), 142.1 (Ph), 140.9 (Ph), 140.1 (Ph), 135.9 (py^4^), 128.0 (Ph), 127.9 (Ph), 127.8 (Ph), 127.4 (d, *J* = 5.4 Hz, Ph), 127.2 (Ph), 126.5 (d, *J* = 9.6 Hz, Ph), 126.3 (Ph), 126.1 (Ph), 126.0 (Ph), 114.1 (${\text{C(CH}}_{3} )_{2}^{\text{TAD}}$), 97.0 (py^3,5^), 87.5 (t, *J* = 8.5 Hz, C(Ph)_2_), 86.2 (C(Ph)_2_), 78.9 (CH^TAD^), 78.4 (CH^TAD^), 25.8 (${\text{CH}}_{3}^{\text{TAD}}$), 25.2 (${\text{CH}}_{3}^{\text{TAD}}$) ppm; ^31^P{^1^H} NMR (CD_2_Cl_2_, 20 °C): *δ* = 197.8 ppm; IR (ATR): $\bar{\nu }$ = 1974 (*ν*_CO_), 1921 (*ν*_CO_), 1865 (*ν*_CO_) cm^−1^.

#### (*N*^2^,*N*^6^-bis((3a*R*,8a*R*)-2,2-dimethyl-4,4,8,8-tetraphenyltetrahydro[1,3]dioxolo[4,5-*e*][1,3,2]dioxaphosphepin-6-yl)pyridine-2,6-diamine)(tricarbonyl)molybdenum(0) ([Mo(PNP-TADDOL)(CO)_3_], **4**, C_70_H_61_MoN_3_O_11_P_2_)

The product was obtained as a yellow solid in 90% yield. ^1^H NMR (CD_2_Cl_2_, 20 °C): *δ* = 7.57 (d, *J* = 7.3 Hz, 4H, Ph), 7.44–7.39 (m, 4H, Ph), 7.34 (vt, *J* = 7.4 Hz, 4H, Ph), 7.30 (vt, *J* = 7.2 Hz, 2H, Ph), 7.26–7.11 (m, 26H, Ph), 6.90 (t, *J* = 7.9 Hz, 1H, py^4^), 5.34 (dd, *J* = 36.4, 7.9 Hz, 4H, CH^TAD^), 5.27 (d, ^3^*J*_HH_ = 8.0, 2H, py^3,5^), 5.09 (s, 2H, NH), 0.50 (s, 6H, ${\text{CH}}_{3}^{\text{TAD}}$), 0.40 (s, 6H, ${\text{CH}}_{3}^{\text{TAD}}$) ppm; ^13^C{^1^H} NMR (CD_2_Cl_2_, 20 °C): *δ* = 221.6 (t, *J* = 8.1 Hz, CO), 206.7 (t, *J* = 13.0 Hz, CO), 155.4 (t, *J* = 10.3 Hz, py^2,6^), 144.2 (Ph), 141.5 (Ph), 140.9 (Ph), 140.0 (Ph), 136.3 (py^4^), 127.9 (Ph), 127.8 (Ph), 127.6 (Ph), 127.3 (d, *J* = 12.9 Hz, Ph), 127.1 (Ph), 126.4 (d, *J* = 10.6 Hz, Ph), 126.2 (Ph), 126.1 (Ph), 126.0 (Ph), 114.2 (${\text{C(CH}}_{3} )_{2}^{\text{TAD}}$), 97.6 (py^3,5^), 87.9 (t, *J* = 7.9 Hz, C(Ph)_2_), 85.9 (C(Ph)_2_), 78.7 (d, *J* = 27.6 Hz, CH^TAD^), 25.7 (${\text{CH}}_{3}^{\text{TAD}}$), 25.3 (${\text{CH}}_{3}^{\text{TAD}}$) ppm; ^31^P{^1^H} NMR (CD_2_Cl_2_, 20 °C): *δ* = 172.7 ppm; IR (ATR): $\bar{\nu }$ = 1980 (*ν*_CO_), 1946 (*ν*_CO_), 1867 (*ν*_CO_) cm^−1^.

#### (*N*^2^,*N*^6^-bis((3a*R*,8a*R*)-2,2-dimethyl-4,4,8,8-tetraphenyltetrahydro[1,3]dioxolo[4,5-*e*][1,3,2]dioxaphosphepin-6-yl)pyridine-2,6-diamine)(tricarbonyl)tungsten(0) ([W(PNP-TADDOL)(CO)_3_], **5**, C_70_H_61_WN_3_O_11_P_2_)

The product was obtained as a yellow solid in 89% yield. ^1^H NMR (CD_2_Cl_2_, 20 °C): *δ* = 7.58 (d, *J* = 7.2 Hz, 4H, Ph), 7.42 (d, *J* = 7.5 Hz, 4H, Ph), 7.38–7.35 (m, 4H, Ph), 7.32 (d, *J* = 7.9 Hz, 2H, Ph), 7.25–7.16 (m, 26H, Ph), 6.88 (t, *J* = 7.9 Hz, 1H, py^4^), 5.35 (dd, *J* = 27.7 Hz, 7.9 Hz, 4H, CH^TAD^), 5.30 (d, ^3^*J*_HH_ = 8.0, 2H, py^3,5^), 5.09 (d, ^2^*J*_PH_ = 8.0 Hz, 2H, NH), 0.50 (s, 6H, ${\text{CH}}_{3}^{\text{TAD}}$), 0.41 (s, 6H, ${\text{CH}}_{3}^{\text{TAD}}$) ppm; ^13^C{^1^H} NMR (CD_2_Cl_2_, 20 °C): *δ* = 214.3 (br, CO), 201.0 (t, *J* = 9.3 Hz, CO), 157.4 (t, *J* = 10.6 Hz, py^2,6^), 145.0 (Ph), 142.4 (Ph), 141.9 (Ph), 140.9 (Ph), 137.4 (py^4^), 128.8 (Ph), 128.8 (Ph), 128.5 (Ph), 128.4 (d, *J* = 12.2 Hz, Ph), 128.1 (Ph), 127.5 (d, *J* = 10.3 Hz, Ph), 127.2 (Ph), 127.1 (Ph), 127.0 (Ph), 115.2 (${\text{C(CH}}_{3} )_{2}^{\text{TAD}}$), 97.8 (py^3,5^), 89.0 (t, *J* = 7.9 Hz, C(Ph)_2_), 87.1 (C(Ph)_2_), 79.7 (d, *J* = 37.2 Hz, CH^TAD^), 26.7 (${\text{CH}}_{3}^{\text{TAD}}$), 26.2 (${\text{CH}}_{3}^{\text{TAD}}$) ppm; ^31^P{^1^H} NMR (CD_2_Cl_2_, 20 °C): *δ* = 153.8 (^1^*J*_w–p_ = 498.8 Hz) ppm; IR (ATR): $\bar{\nu }$ = 1978 (*ν*_CO_), 1932 (*ν*_CO_), 1865 (*ν*_CO_) cm^−1^.

### X-ray structure determination

X-ray diffraction data of **4**, in the form of the methylene chloride solvate **4**·3CH_2_Cl_2_ (CCDC 1845599), were collected at *T* = 100 K in a dry stream of nitrogen on a Bruker Kappa APEX II diffractometer system using graphite-monochromatized Mo *K*α radiation (*λ* = 0.71073 Å) and fine sliced φ- and ω**-**scans. Data were reduced to intensity values with SAINT and an absorption correction was applied with the multi-scan approach implemented in SADABS [37]. The structure was solved by the dual-space approach implemented in SHELXT [38] and refined against *F*^2^ with SHELXL [39]. Non-hydrogen atoms were refined anisotropically. The H atoms connected to C atoms were placed in calculated positions and thereafter refined as riding on the parent atoms. The amine-hydrogen atoms were located from difference Fourier maps and refined freely. The absolute structure was confirmed by resonant scattering [Flack parameter 0.025(14)]. Important crystallographic data are: C_70_H_61_MoN_3_O_11_P_2_ 3(CH_2_Cl_2_), *M*_r_ = 1532.87, yellow plates, 0.50 × 0.25 × 0.04 mm, monoclinic, space group *P*2_1_ (no. 4), *a* = 10.0035(8) Å, *b* = 20.2073(18) Å, *c* = 18.1231(16) Å, *β* = 103.943(4)°, *V* = 3555.5(5) Å^3^, *Z* = 2, *μ *= 0.517 mm^−1^, *d*_x_ = 1.432 g cm^−3^. 20,807 reflections were collected up to *θ*_max_ = 30.0°; *R*_1_ = 0.0548 (14,397 reflections with *I* > 2*σ*(*I*)), *wR*_2_ = 0.1072 (all data), 877 parameters.
